# The Role of the Surgeon in the Germline Testing of the Newly Diagnosed Breast Cancer Patient

**DOI:** 10.3390/curroncol30050353

**Published:** 2023-05-01

**Authors:** Stephanie Schick, Joshua Manghelli, Kandice K. Ludwig

**Affiliations:** Department of Surgery, School of Medicine, Indiana University, Indianapolis, IN 46202, USA

**Keywords:** breast cancer, germline testing, breast surgeon, genetic mutations, genetic counseling

## Abstract

For patients with newly diagnosed breast cancer, information regarding hereditary predisposition can influence treatment decisions. From a surgical standpoint, patients with known germline mutations may alter decisions of local therapy to reduce the risk of second breast primaries. This information may also be considered in the choice of adjuvant therapies or eligibility for clinical trials. In recent years, the criteria for the consideration of germline testing in patients with breast cancer has expanded. Additionally, studies have shown a similar prevalence of pathogenic mutations in those patients outside of these traditional criteria, prompting calls for genetic testing for all patients with a history of breast cancer. While data confirms the benefit of counseling by certified genetics professionals, the capacity of genetic counselors may no longer meet the needs of these growing numbers of patients. National societies assert that counseling and testing can be performed by providers with training and experience in genetics. Breast surgeons are well positioned to offer this service, as they receive formal genetics training during their fellowship, manage these patients daily in their practices, and are often the first providers to see patients after their cancer diagnosis.

## 1. Introduction

Approximately 10% of breast cancers are associated with a pathogenic germline mutation [1]. For women and men with newly diagnosed early-stage breast cancer, information regarding their hereditary predisposition can influence treatment decisions. From a surgical standpoint, patients with known mutations may alter their decisions regarding local therapy to reduce the risk of second breast primaries. Such genetic information may also be considered in the choice of adjuvant therapies or eligibility for clinical trials.

In recent years, the National Comprehensive Cancer Network (NCCN) has expanded its criteria for the consideration of germline testing in patients with breast cancer. Additionally, studies have shown a similar incidence of pathogenic mutations in women outside these traditional criteria, prompting calls for testing for all patients with a history of breast cancer. While data confirms the benefit of genetic counseling, the model of testing by certified genetic counselors (CGC) may no longer meet the needs of these growing numbers of patients. This manuscript will further discuss the role of the surgeon in the genetic testing of the breast cancer patient.

## 2. Materials and Methods

We performed a comprehensive literature search using the PubMed database (up to 1 February 2023) of the following keywords in the title or abstract (germline testing, hereditary breast cancer, genetic testing, genetic counseling, *BRCA*, *CHEK2*, *PALB2*, and *TP53*). The types of articles reviewed included: systematic reviews and meta-analyses, institutional series, cohort studies, national practice guidelines, and consensus statements in the English language. Additional pertinent articles were chosen based on clinical experience, reviews of the bibliography of these articles or to address specific scenarios. Articles were included if they addressed the following research questions: the prevalence of germline mutations in newly diagnosed breast cancers; the differences in outcomes and treatment in mutation carriers vs sporadic controls; the current state in the delivery of pre- and post-test counseling and disparities in access of certified professionals; and the role of the surgeon in providing germline testing. In total, 61 studies were included in this review.

## 3. Results

### 3.1. The Prevalence of Germline Mutations in Breast Cancer Patients

Approximately 10% of breast cancers are associated with a hereditary predisposition and evidence-based guidelines have been established by the NCCN to guide clinicians regarding eligible candidates for testing [1,2]. The most recent criteria have been updated to include young age at diagnosis, Ashkenazi Jewish ancestry, triple negative subtype, and significant family history of breast, ovarian, or other associated cancers (Table 1) [2]. Historically, germline testing consisted of a selection of individual genes based on a review of relevant family history, but the introduction of next-generation platforms has enabled the sequencing of multiple genes simultaneously at a lower cost. A benefit of larger panel testing is the increased identification of moderate penetrance genes that may alter clinical decision-making, while limitations include higher rates of variants of uncertain significance (common) or findings that lack management guidelines (rare) [3].

**Figure 1:**
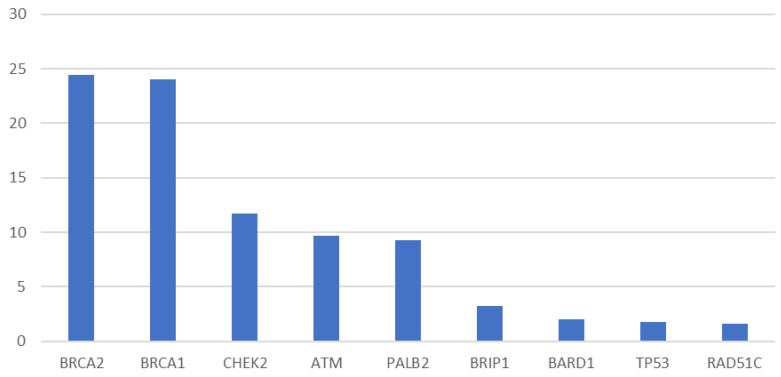
The percentage of pathogenic variants identified on a 25-Gene Panel in women with breast cancer [5]. The figure in the initial publication by Buys et al included NBN as it was included in the multi-gene panel. The authors have removed it from the chart as population-based analyses have shown no association with breast cancer risk [7].

Multiple studies have evaluated the prevalence of pathogenic or likely pathogenic variants (PV or LPV) in women with breast cancer, although the majority are limited to retrospective analyses from single institutions or laboratories. Tung et al published a review of comprehensive panel results from 488 women from a single academic institution with non-metastatic breast cancer. In their cohort, the overall prevalence of PV or LPV was 10.7%, with *BRCA* mutations identified in 57%, followed by *CHEK2* (19%), *ATM* (7.6%), and *BRIP1* (7.6%) [4]. Similar findings were demonstrated by two industry-sponsored analyses of multi-gene panel testing in 35,409 women with a single diagnosis of breast cancer. Pathogenic variants were found in 9–10% of patients, with *BRCA* mutations most common (48%), followed by *CHEK2*, *ATM*, and *PALB2* (Figure 1) [4,5,6].

Using the Georgia-California SEER Genetic Testing Linkage Initiative, Kurian and colleagues evaluated the test results from 77,085 patients with breast cancer, as tested by 4 separate laboratories. In their cohort, the prevalence of PV or LPV was 7.8%, and the distribution of mutations was similar to the above studies [8]. The authors also sought to determine the factors associated with the presence of PV or LPV; while the factors of younger age at diagnosis and triple negative subtype have consistently been associated with an increased risk of a *BRCA* mutation, no other factors were associated with other genes [4,5,6,8].

Like female breast cancers, most studies evaluating mutations in male breast cancer are retrospective analyses from single institutions or laboratories. The prevalence of PV or LPV in men with breast cancer ranges from 132%, with *BRCA2* being the most common [9,10]. Mutations in *BRCA1* can be identified, but less frequently than in *BRCA2*. In those who test negative for PVs in *BRCA*, the next most common mutations were *CHEK2* and *PALB2*, demonstrating the need for multi-gene panel testing (Table 2) [11]. The age at diagnosis does not appear to be associated with PV [10]. This higher prevalence of germline mutations has prompted the NCCN to recommend germline testing for all men with breast cancer regardless of age [2].

It is not uncommon for women with breast cancer who are outside the traditional criteria to request testing. While it is feasible, the cost of testing may not be reimbursed by insurance if they do not meet the criteria. Two industry-sponsored studies evaluated the presence of PV or LPV in women with breast cancer outside of the NCCN guidelines. In a cohort of 4196 Medicare patients who underwent comprehensive panel testing by a sole laboratory, similar rates of PV or LPV were identified, whether patients met the criteria or not (10.5% vs. 9%, *p* = 0.26) [12]. Comparable findings were noted in a multicenter cohort of 959 women with a personal history of breast cancer tested with multi-gene panels by a single laboratory. For those within the criteria, the likelihood of PV or LPV was 9.4%, compared to 7.9% outside of the criteria (*p* = 0.42). Not surprisingly both studies demonstrated fewer *BRCA* mutations in patients outside of the criteria, as the NCCN guidelines were originally established to identify *BRCA* mutations [6,12]. These studies reporting similar rates of the prevalence of germline mutations in those women outside of the traditional criteria have led some to call for universal testing for all women with a history of breast cancer, including the American Society for Breast Surgeons (ASBrS) [13]. A third study performed by the Mayo Clinic evaluated the prevalence of mutations in 3907 women with a personal history of breast cancer tested at their institution. In their cohort, there was a lower prevalence of PV or LPV in women outside of the NCCN criteria (9.0% vs. 3.5%, *p* < 0.001), but they also noted that 29.9% of women with pathogenic mutations identified in a 9-gene panel would not have qualified for testing per the NCCN. Younger age at diagnosis improved the sensitivity of testing, so the authors proposed a hybrid testing model that utilizes the NCCN guidelines for women over 65 and the consideration of testing for all women with breast cancer under the age of 65 [14].

### 3.2. The Benefits of Testing—How Does Genetic Testing Alter Clinical Decision-Making?

#### 3.2.1. Local Therapy Decisions

Should women with known PV or LPV avoid breast conservation therapy (BCT) when diagnosed with breast cancer? There are no randomized trials to address this question. Since these pathogenic variants are rare, data evaluating the risk of local recurrence (LR) after BCT in germline mutation carriers are limited and observational.

***BRCA*.** As *BRCA1* and *BRCA2* were the first genes identified in hereditary breast cancer syndromes, outcomes in these patients are most reported. A meta-analysis of 10 studies comparing BCT in *BRCA* carriers to sporadic controls demonstrated a slight increase in LR (17.3% vs 11.0%, *p* = 0.07), but this was not statistically significant. The authors concluded it was likely a result of new primaries rather than true LR, as in those studies with a longer follow-up (>7 years), there was a significant increase [15]. A separate meta-analysis published in 2019 of 19 studies comparing BCT to mastectomy in *BRCA* carriers reported a higher LR at 5, 10, and 15-year follow-ups. However, in the BCT group, the risk of LR consistently increased over time, while in the mastectomy group, the risk of LR did not appear to increase after 5 years, again suggesting this was a result of second breast primaries [16]. Furthermore, studies evaluating the toxicity and risk of contralateral breast cancer (CBC) show no difference in *BRCA* carriers receiving definitive radiation for their index breast cancer. Therefore, one can conclude that *BRCA* carriers should be considered for BCT if otherwise eligible, but counseling regarding the risk of second breast primaries and enhanced surveillance with annual mammography and breast MRI should be performed [17].

***TP53*.** Breast cancer is one of the most common cancers associated with TP53 mutations (aka Li-Fraumeni Syndrome or LFS), with 85% of women developing cancer before age 60. This mutation is extremely rare, with <1% prevalence on multi-gene panel testing in women with breast cancers [18]. Few studies exist that question whether this mutation is associated with increased LR. A retrospective Chinese study noted a significant increase of in-breast recurrence after BCT in *TP53* carriers when compared to sporadic controls (median 6.7 years follow-up, 21.3% vs. 3.8%, *p* = 0.006) with no associated increase after mastectomy. Looking specifically at the TP53 carriers, the risk of recurrence was higher after BCT when compared to mastectomy (21.1% vs. 0%, *p* = 0.038) [19]. In addition to concerns about LR, retrospective small cohort studies reported rates of radiation-induced secondary cancers as high as 30% in patients with LFS, questioning the safety of radiotherapy and leading to the recommendation of mastectomy for this population [20,21]. The largest series to address this question was recently reported by Le et al, comparing 18 patients with LFS to 3731 women without LFS who underwent definitive radiation for their breast cancer. In the LFS group, at a median 12.5-year follow-up, one patient developed thyroid cancer (6%) and one developed a sarcoma within the radiated field (6%). In the sporadic controls at median 4-year follow-up, 17 patients developed thyroid cancer (0.5%) and one patient developed a sarcoma (0.03%). Propensity score analysis demonstrated a significant increase in thyroid cancers (RR 23.8, *p* = 0.001), and a non-significant increase in sarcomas (RR 5.9, *p* = 0.22) after radiation for breast cancer in patients with LFS. The authors concluded that the risk of radiation-induced cancers may not be as high as previously reported, but decisions regarding the use of radiotherapy should be individualized [22].

**Moderate penetrance genes.** Data regarding clinical outcomes are lacking in moderate penetrance genes such as *ATM*, *PALB2*, or *CHEK2*. Small cohort studies demonstrate no difference in LR or second breast primaries after BCT when compared to women without these mutations [23]. Additionally, like *BRCA* carriers, there is no increase in toxicity or CBC after definitive radiation in these patients [24,25]. There is one exception in young women with rare missense mutations in ATM who may have an increased risk of CBC after radiation to the affected breast, but these mutations are extremely rare and whether they are pathogenic is in question [26]. Therefore, for carriers of these moderate penetrance genes, locoregional therapy decisions regarding the use of BCT should be approached similarly to non-mutation carriers [27].

#### 3.2.2. The Risk of Contralateral Breast Cancer 

Multiple studies have suggested that women with germline PV or LPV are at an increased risk of CBC, but like studies evaluating LR, data regarding CBC is limited. A recent publication by Yadav et al estimated the risk of CBC in PV carriers as compared to sporadic controls as a part of the Cancer Risk Estimates Related to Susceptibility (CARRIERS) consortium. In this prospective review of 15,104 women with breast cancer with 11-year median follow-up, CBC was seen in 5.3%, with a significant increase in women with *BRCA1*, *BRCA2*, and *CHEK2* mutations (*p* < 0.05). Hazard ratios were as follows: *BRCA1* 2.7, *BRCA2* 3.0, and *CHEK2* 1.9. There was no overall increase in CBC in women with *PALB2* or *ATM* mutations; however, a significant increase was seen in *PALB2* carriers with ER-negative tumors (HR 2.9, *p* = 0.006). In all groups, the age at diagnosis was the strongest predictor, with an increased risk of CBC after menopause only seen in *BRCA2* carriers (HR 3.0, *p* < 0.001) [28]. These findings are consistent with other retrospective studies [29,30,31,32]. As for women with TP53 mutations, the specific risk of CBC is 17.9% at 10 years [21].

Knowledge regarding the risk of CBC allows women to consider contralateral prophylactic mastectomy (CPM) for risk reduction and the avoidance of imaging surveillance. Van Sprundel et al investigated the effect of CPM in *BRCA* carriers with a personal history of invasive breast cancer. At mean the 5-year follow-up, CPM reduced the risk of a CBC by 91%, although there were no statistically significant differences in the overall survival between the CPM and surveillance groups [33]. These results were redemonstrated in an updated Cochrane review published in 2018 [34]. More data is needed to determine the role of CPM in women with moderate penetrance genes. The mutation status alone should not determine the role of CPM; other factors such as age at diagnosis, the overall prognosis from cancer diagnoses, competing comorbidities, and the ability to undergo imaging surveillance should be considered [18].

For women with newly diagnosed breast cancer, testing prior to surgery is beneficial as it provides the opportunity to assess the risk of local recurrence and additional breast primaries when making decisions about surgery. Multiple studies have shown that knowledge of the PV or LPV status is associated with bilateral mastectomy rates, and for those who test negative, reassurance can be provided. Kurian et al reported the use of bilateral mastectomies in 61.7% of *BRCA* carriers, 42.5% with other gene mutations, and 24.3% of women with negative testing results [35]. Chiba and colleagues reviewed the timing of genetic testing for 173 BRCA carriers and its impact on the choice of surgery. For those who knew their results prior to surgery, 86% underwent bilateral mastectomies, compared to 29% of those identified after surgery (*p* < 0.001). Furthermore, of those identified to be carriers after surgery was complete, 59% elected to undergo delayed bilateral mastectomies [36].

#### 3.2.3. The Impact on Systemic Therapy

The identification of germline mutations may allow for additional options for systemic therapy, such as molecularly targeted agents. For women with metastatic disease, two large, randomized, open-label, phase 3 trials (OlympiAD and EMBRACA) demonstrated significant benefits in progression-free survival and improved the quality of life with poly (ADP-ribose) polymerase (PARP) inhibitors (olaparib or talazoparib) in BRCA carriers [37,38,39]. In the adjuvant setting, the OlympiA trial evaluated the use of olaparib for 1836 women with germline BRCA mutations with high-risk early-stage breast cancer. In this trial, olaparib was associated with improved disease-free (HR 0.58, *p* < 0.0001) and overall survival (HR 0.68, *p* < 0.009), prompting the Food and Drug Administration to approve it for use in this setting in March 2022 [40]. The findings of these studies have prompted an addition to the NCCN guidelines to include the consideration of testing for those patients for whom testing may aid in systemic therapy [2]. Although the other moderate penetrance genes are in the same DNA damage response pathway as BRCA1 and BRCA2, more investigation is needed to determine the role of these targeted agents in patients with other germline mutations [18].

## 4. Discussion

For the reasons listed above, testing for germline mutations has become a key component of breast cancer treatment decisions. One of the most important aspects of this algorithm includes the pre- and post-test counseling and documentation of this may be mandated for insurance coverage. These discussions should aim to reduce distress and help the patient understand the benefit of testing as a vehicle to obtain information about personal risk, empowering the patient to consider interventions to manage or reduce that risk [13]. A Polish study of 3524 women undergoing BRCA testing noted a correlation between patient-reported high levels of anxiety prior to testing and sustained high levels of anxiety after testing. When considering testing, a brief psychological assessment can be performed to identify patients at higher risk [41]. Pre-test counseling should also include a three-generation pedigree to determine the choice of panel based on family history. Once the decision to proceed with testing has been made, informed consent should include the benefits and limitations of testing, including the possibility of inconclusive results, incorrect perception of risk, and discriminatory ramifications. It is important to carefully review potential results such as PV or LPV, negative or benign, or variant of unknown significance (VUS) [13]. The use of multigene panels is associated with the identification of VUS in 18–54% of patients [3,4,5,6,8].

Post-test counseling primarily focuses on the interpretation and delivery of test results, either in person, by phone, or virtually. For patients with negative results or VUS, it is recommended that medical decisions should be made based on treatment-related factors and personal and family histories of cancer [42]. For those with PV or LPV results, the current evidence for that specific mutation should be reviewed to develop a customized plan for the management of the current breast cancer. These patients are often at a higher risk for other primaries outside the breast, so referrals to specialists to discuss surveillance and risk reduction should be made. Moreover, it is also important to discuss cascade testing for family members [13,43]. Lastly, studies such as the Lifestyle Intervention Study in Women with Hereditary Breast and Ovarian Cancer (LIBRE) demonstrated that higher levels of physical activity and the avoidance of smoking were associated with significantly lower incidences of cancer in BRCA carriers [44]. These recommendations are appropriate lifestyle modifications for all patients regardless of germline testing results [13].

The gold standard would be for counseling to be provided by an expert in cancer genetics [2]. Stenehjem et al demonstrated a 6.3-fold higher rate of completion of testing in patients who underwent counseling by a CGC [45]. After testing is complete, the involvement of a provider with specialized training allows for the accurate interpretation of testing results, patient education, and the ordering of appropriate follow-up tests and referrals. However, as noted by the panel at the time of the 2020 update to the NCCN guidelines, “most genetic testing is conducted by providers with limited expertise in genetics, and often without pre-test genetic counseling” [2]. With the expansion of criteria for those to be considered for testing, it is not feasible for all to see a CGC. While the profession has grown significantly in the last 10 years, there is still a shortage. As reported by the National Society of Genetic Counselors, there were 5,629 CGCs in the United States in 2021, with the majority practicing outside the realm of cancer genetics (57%). Additionally, there is a heavy imbalance in the geographic distribution of CGCs, with 49% of the workforce practicing in only 10 states [46].

Alternative models have been investigated to improve access to CGCs. Since the need for physical examination is limited, genetic counseling is well-suited for remote delivery by telephone or videoconference, removing the barriers of travel, time off work, and the need for childcare. A meta-analysis of 3 randomized studies of 2753 patients undergoing BRCA testing showed that telephone counseling was not inferior to in-person visits in patient-reported cancer-specific distress or genetics knowledge, and the authors concluded that telephone consults may be considered as an alternative method to improve access [47]. However, as reported by surveys of CGCs, these platforms can be limited by the inability to detect non-verbal cues and may not be appropriate for those with hearing, visual, or language difficulties. There may also be issues with reimbursement [48]. More recently, the COVID pandemic forced a significant increase in the utilization of telehealth platforms across all specialties, which allows for face-to-face interaction and screen-sharing for the utilization of resources. Investigators at the University of Pennsylvania noted a significant increase in both counseling and testing with the utilization of telehealth in community hospitals where CGCs were not available on-site (OR 30.5, *p* < 0.001) [49]. A systematic review of 10 studies evaluating the use of videoconferencing for genetic counseling showed similar patient-reported levels of satisfaction, cancer-related distress and knowledge gained when compared to in-person consultations [50]. While there are benefits to telehealth, it does require a working device, stable internet connection and technical ability to access the platform, which may be a barrier for patients with lower socioeconomic status or older patients. As this technology is still relatively new and evolving, more data is needed to establish the long-term role of telehealth in genetic counseling.

Telehealth and phone consultations avoid the need for in-person appointments, but the inconvenience of another appointment to meet with a CGC could adversely result in delays in definitive treatment or the failure to have testing at all. Ochoa et al performed a retrospective review of the referral process for genetic counseling in women under treatment for breast cancer at their institution. Of the 392 women referred, 20.9% did not pursue scheduling a consultation, and another 19% either cancelled or failed to show up for the appointment. For those whose results would impact treatment plans, the time from referral to consultation was an average of 1.7 months, delaying definitive surgical therapy decisions [51]. Others have shown that the introduction of mandates for consultation with CGCs by insurance companies resulted in significantly fewer tests completed [45].

Point-of-care testing in the context of existing appointments can improve access. Culver and colleagues reported 95% acceptance of counseling and 93% completion of testing when all patients seen in a multidisciplinary breast cancer clinic were offered an immediate consult with a CGC [52]. These findings are similar to the results reported in other types of cancer [53,54]. While this makes sense, having a CGC in the clinic may not be feasible due to a lack of available workforce, physical space, or funding. Several national organizations assert that genetic counseling and testing could be provided by other healthcare professionals with specialized training in genetics, including the American Society for Clinical Oncology (ASCO), American College of Surgeons Committee on Cancer and the National Accreditation Program for Breast Centers [54,55,56]. Pathways to streamline testing can be created for non-genetic clinicians with the input of CGCs. Ain et al performed a retrospective analysis of 474 women with newly diagnosed breast cancers tested via a mainstream genetic pathway at their institution. The median time from testing to result was 18 calendar days (range 15–21 days), and the authors noted timely turnaround of results was associated with a significant reduction in bilateral mastectomy rates (*p* < 0.0001) [57].

This is where breast surgeons can serve an important role. Breast surgeons receive a formal education regarding cancer genetics during fellowship training, and routinely manage carriers of mutations as a part of daily practice. A 2014 survey of practicing breast surgeons reported that the majority routinely performed the key components of counseling and testing—obtaining a 3-generation family pedigree, counseling of risks and benefits of testing, and interpreting and discussing results [58]. They are often the first provider to see patients after their diagnoses, direct locoregional therapy decisions, and can counsel the patients on how testing results will influence therapeutic options for breast cancer. The surgeon can obtain testing as part of the initial cancer consultation, avoiding the need for additional appointments and eliminating delays in result turnaround. Since the majority of patients test negative, this would allow access to a CGC to be reserved for patients with PV or LPV. While the surgeon may not directly manage other cancer risks, they are well positioned to make referrals to other specialists to address surveillance and risk reduction strategies.

Several educational programs have been developed to educate surgeons and non-genetic healthcare providers with an interest in genetic counseling and testing. Societies such as ASCO and the ASBrS have developed courses at the time of annual meetings for providers seeking additional education [59,60]. Other options include institution-based intensive courses and less formal webinar series that provide up-to-date information [61]. When choosing a course, one should look for topics that include criteria for testing, consideration of the choice of panel, informed consent, insurance issues, test interpretation, post-test counseling and management, and discussions regarding cascade testing for family members. When patients are found to carry PV or LPV mutations, referral to a CGC should be considered when available. For those without access to CGCs, there are several resources available to assist in gathering the appropriate information to help guide discussions regarding the assessment of risk, surveillance and screening, and strategies for risk reduction.

## 5. Conclusions

The role of germline testing in newly diagnosed breast cancer patients continues to evolve. The number of patients who are candidates for testing continues to expand, with some calling for testing in all women with breast cancer. While the benefits of counseling by a certified genetics professional are well-established, unfortunately, there is an inadequate workforce to accommodate this need. Breast surgeons can help bridge this gap. To provide adequate genetic counseling, however, it is important for surgeons to have experience in identifying patients who are candidates for testing, perform informed consent and pretest counseling, provide post-test counseling with a discussion of management strategies, and refer family members for cascade testing when appropriate. There are various tools that can assist in calculating risk as well as management strategies for surveillance and risk reduction.

## Figures and Tables

**Table 1:** The relevant criteria for the consideration of germline testing for newly diagnosed breast cancers per NCCN Guidelines (v. 3.3023) Adapted with permission from [2]. 2023, National Comprehensive Cancer Network, Inc.

Individual with blood relative with PV/LPV in cancer susceptibility gene
Individuals who previously tested negative by limited testing panel
Breast cancer diagnosis ≤ age 50
Male breast cancer regardless of age
Triple negative histology regardless of age
Ashkenazi Jewish ancestry regardless of age
Lobular histology with personal or family history of diffuse gastric cancer
Multiple breast primaries
High risk cancers who may be considered candidates for molecular targeted agents
Metastatic breast cancers
Personal history of breast cancer with close family members with breast cancer ≤ age 50, ovarian cancer, prostate cancer, male breast cancer, pancreatic cancer

These are the most recent guidelines at the time of the manuscript submission and are updated annually. These can be accessed via the NCCN website.

**Table 2:** The most common pathogenic mutations in men with breast cancer using multi-gene panel testing [10,11].

*BRCA2*	23.1%
*BRCA1*	4.6%
*CHEK2*	2.4%
*PALB2*	1.8%
*ATM*	1.2%
